# Perception of robotic actions and the influence of gender

**DOI:** 10.3389/fpsyg.2024.1295279

**Published:** 2024-01-31

**Authors:** Miriam Abel, Giovanni Buccino, Ferdinand Binkofski

**Affiliations:** ^1^Special Education and Rehabilitation of Speech and Language Disabilities, Department of Rehabilitation and Special Education, Faculty of Arts and Humanities, University of Cologne, Cologne, Germany; ^2^Department of Neuroscience, IRCCS Istituto Scientifico San Raffaele, Università Vita Salute San Raffaele, Milan, Italy; ^3^Research Center Juelich GmbH, Institute for Neuroscience and Medicine (INM-4), Juelich, Germany; ^4^Division for Clinical Cognitive Sciences, University Hospital RWTH Aachen, Aachen, Germany

**Keywords:** action observation system, mirror neuron system (MNS), perception of robotic movements, perception of humanoid movements, gender influence on perception of robotic movements

## Abstract

In our society interaction with robots is becoming more and more frequent since robots are not only used in the industry, but increasingly often in assistance and in health system. Perception of robots and their movements is crucial for their acceptance. Here we shortly review basic mechanisms of perception of actions, and then of perception of robotic and human movements. The literature demonstrates that there are commonalities, but also differences in the perception of human and robotic movements. Especially interesting are biologic gender differences in the perception of robotic movements. The results show that males seem to be more sensitive to the differences between robotic and anthropomorphic movements, whereas females seem not to perceive such differences. However, females transfer more anthropomorphic features to robotic movements. While looking at the brain activation during perception of humanoid and robotic movements in different genders one can conclude that different strategies are used; female seem to analyse robotic movements online, while male seem to use previous knowledge from interaction with robots. Further research is needed to specify more such gender differences.

## Action perception and the mirror neurons system

Action perception has gained much attention during the last decades. The so-called mirror neuron system (MNS) has been recognized as the major player in action perception, action understanding and action imitation (Rizzolatti and Craighero, 2004). The MNS includes the ventral premotor cortex (vPMC), inferior parietal cortex (IPC), the supplementary motor area (SMA) and to some extent the superior temporal cortex (Di Pellegrino et al., 1992; Gallese et al., 1996; Rizzolatti and Craighero, 2004; Fogassi et al., 2005; Kaysers and Gazzola., 2010; Mukamel et al., 2010). Areas within the MNS have been found to be involved in both performing a specific goal-directed action and observing/recognizing the same or a similar action, when performed by another individual (Di Pellegrino et al., 1992; Gallese et al., 1996; Rizzolatti and Craighero, 2004). At least in humans, there is evidence that areas within the MNS are also involved in motor imagery, when individuals are requested to imagine goal directed actions in the absence of any motor output, and language processing, when individuals attribute meanings to read or heard language items, especially expressing a motor content (Buccino et al., 2016; Hardwick et al., 2018). As a whole, experimental evidence suggest that the MNS is responsible for the matching of sensory information (visual, acoustic) about motor actions with the motor representations of those actions in the perceiver’s brain. In this respect, one can also speak of the translation of a perceived action into one’s own motor processes. According to current studies, this should make it possible to assess both the further actions and the intention of the counterpart. In particular, it was found that the MNS is more activated when the observed movement is familiar to the motor repertoire of the beholder (Buccino et al., 2004a). For example, in a fMRI study it has been shown that the MNS becomes active when human individuals observe an action like biting, regardless of the acting individual (a human individual, a monkey or a dog). This because biting is an action shared with other species. In contrast with this, when human individuals observe actions like silent speech, barking or lip-smacking (an action that monkeys use in an affiliative manner), then only silent speech recruits the sensorimotor areas of the MNS. No recruitment occurs during the observation of lip-smacking or barking. Indeed, these are actions that are not part of the human motor repertoire and hence not shared. This evidence suggests that a direct experience and a motor competence of the observed actions are fundamental pre-requisites for the MNS to be recruited. In a similar vein, Calvo-Merino et al. (2005) found a stronger involvement of the MNS in the observation of capoeira dancing movements by capoeira dancers than by classical ballet dancers and vice versa. Similar results were obtained by Haslinger et al. (2005) when studying the perception of piano players. Again, higher MNS activations were found when the observing subjects were themselves professional piano players as opposed to amateur players. In summary, actions that are part of the observer’s motor repertoire are mapped onto his or her own motor system and stimulate stronger activations in the MNS. Actions that are not part of the repertoire seem to excite the motor system less and elicit lower activations in the MNS. Complementary to this evidence, when human individuals have to learn novel actions (think of learning how to play a musical instrument, to dance and so on) or face new situations, besides and beyond the MNS, other areas in the brain become active. In an fMRI study (Buccino et al., 2004b), when naïve participants had to learn some guitar accords presented through a videoclip by an expert guitarist, areas in the prefrontal cortex became active. Interestingly, the prefrontal cortex was not active if the same accords were observed by expert guitarists or even by naïve participants, after an appropriate training (Vogt et al., 2007).

## Perception of robotic actions

It is hard to imagine today’s society without robots. Among other things, they are firmly established in industry, in business, but also in healthcare (Karwowski, 1991; Maier, 2022; Michalos et al., 2022). Nowadays, there are many different types of robots. Among others, there are stationary robots, which are popularly used in industry, autonomous robots, such as toy robots, and humanoid robots. Humanoid robots are most similar in appearance and structure to a human form. However, robots also differ in the way they move. Here, a distinction is made between classical robotic movements and humanoid (anthropomorphic) movements. Anthropomorphic means the simulation of human characters by robots (Duffy, 2003; Kuz et al., 2013). The classical robotic movements are point-to-point movements, whereas the human movements describe a bell shaped trajectories of motion. The anthropomorphic movements follow a digressive (concave) curve whereas, the robotic movement follow a progressive (convex) curve. The type of movement influences the comfort factor of a human interacting with a robot (Huber et al., 2008; Kuz et al., 2013). Hugues et al., 2016 found that the target of a movement could be better predicted when the movement is performed in a human-like manner, so that people in a co-working scenario with a robot feel more confident, less stressed and more willing to work together with the robot. Especially the identification with a robot as a co-worker is based on the attitude toward a robot, technological expertise, and personality (Savela et al., 2021). Dubois-Sage et al. (2023) describe a large interindividual variability in anthropomorphism that depends on age, gender, personality, culture, previous experience with technology, level of education, degree of social isolation, and the developmental type of the individual.

As described above, the mirror neuron system is particularly active in the perception of familiar movements. However, recent studies have also demonstrated that specifically the action observation network, a network of parietal, premotor, and occipitotemporal regions in the human brain, is involved in not only perceiving but also understanding the observed movements of others (Gallese and Goldman, 1998; Gallese et al., 2004) and is closely linked to the mirror neuron system.

Cross et al. (2012) showed that specifically the action observation network is active not only during familiar movements, but also during the observation of robotic movements that do not describe a human trajectory. They investigated the activation of the action observation network when viewing human and robotic movements. They were able to show that the action observation network is active both when observing familiar and unfamiliar movements, independent of the actor of the movement. Further studies could show that the action observation network is particularly activated when observing goal-directed and contextually familiar movements (Hamilton and Grafton, 2008; Liepelt et al., 2010; Ramsey and Hamilton, 2010). Liepelt et al. (2010) examined imitation of movements that were human or non-human. They were able to show that both human and non-human movements were better imitated when they were goal-directed. Ramsey and Hamilton (2010) support these results by showing in an fMRI study activation of the inferior parietal cortex to distinguish the goals of human hand actions. They concluded that the understanding of an action is more important than the form of the acting actor (e.g., human or robot).

The theory of higher involvement of the action observation network and the mirror neuron system in goal-directed movements is supported by the results of other studies showing that when movements were not goal-directed, no difference was found in the perception of robotic or anthropomorphic movements (Gazzola et al., 2007; Chaminade et al., 2010; Urgen, 2015; Hoenen et al., 2016). Gazzola et al. (2007) conducted an fMRI study in which they showed subjects humans and robots performing different movements. They were able to show that the goal of a movement is more important for the activation of the mirror neuron system than the type of gestalt performing the movement. However, it has also been shown that the greater the anthropomorphic features of a movement, the greater the activation of the mirror neuron systems (Krach et al., 2008). Thus, it can be stated that for the activation of the MNS the human resemblance of a movement and the orientation toward a goal is important (Gazzola et al., 2007; Krach et al., 2008; Abel et al., 2020).

## Gender influence on perception of robotic actions

Previous studies investigated the influence of the robot’s gender on how humans perceive the robot. For example, smart home devices, such as Google’s Alexa or Apple’s Siri, have a female identity by being assigned a female voice to convey a sense of comfort and security (Stroessner and Benitez, 2019). In contrast, robots that are meant to appear more sinister or powerful are assigned male names and roles, such as Terminator. Overall, robots with a female figure or face are judged to be warmer (Benitez et al., 2017; Carpinella et al., 2017). Stroessner and Benitez (2019) support this hypothesis by showing that anthropomorphic female robots are judged to be warmer than machine-like robots in a male figure, which are viewed with more of an uncomfortable feeling. In general, anthropomorphic robots are judged to be more competent. However, the Uncanny-Valley-Theory (Mori, 1970) describes an effect that turns to the opposite when a robot has too many anthropomorphic features, i.e., if a robot is assigned too many anthropomorphic features, it is no longer judged as positive or perceived as too real. Many authors discuss the relationship of the degree of anthropomorphism and the acceptance of the service robot (Bartneck et al., 2009; Belanche et al., 2020). Lu et al. (2019) were able to show that customer satisfaction increases when the service robot has a high degree of anthropomorphic characteristics. The service robot is then trusted more and people are more satisfied with the robot’s service. However, the gender of the robot is also a decisive factor, since the same expectations and requirements are placed on it as on a real person. Seo (2022) examined emotional responses to a service robot, varying gender (female/male) and degree of anthropomorphism. He was able to show that gender stereotypes are also present in service robots and that the gender of the robot influences the satisfaction in the interaction. In summary, this means that if the robot takes on a role or position that is otherwise more likely to be performed/occupied by women, it is beneficial if the robot is then also attributed female characteristics to increase user acceptance, familiarity, and likability in the co-working scenario (Seo, 2022).

It is now interesting to investigate to what extent the biological sex of the interacting human also has an influence on the perception of a robot actions. Evidence exists that biological females and males could perceive certain details of robotic actions in a different way (Abel et al., 2020). Furthermore, in the field of human-robot interaction, there is a large debate on the role of sex differences in operators on the perception of robots. It is reported that females generally show more positive attitudes toward anthropomorphic robots (Lee, 2008; Abel et al., 2020, 2022). Males, on the other hand, have more positive attitudes toward classical robots (Cameron et al., 2018). Differences in the perception of robotic and humanoid movements were shown by Abel et al., 2020. They figured out that males seem to be more sensitive to the differences between robotic and anthropomorphic movements, whereas females showed no difference between them. However, females transferred more anthropomorphic features to robotic movements. Abel et al. (2020) investigated in a behavioral experiment the perception of anthropomorphic and robotic movements performed by a robot model and a digital human model in biological females and males. They demonstrated that males were more sensitive to the differences in robotic and anthropomorphic movements than women. Whereas women attributed more anthropomorphic characteristics to the robotic movements. These differences were found independent of the kind of actor that performed the movements. To date, there has been little investigation of neuroanatomical differences between males and females in human-robot interaction. A further study by Abel et al., 2022 investigated the neuroanatomical correlates in perception of robotic and anthropomorphic movements between biological males and females. In an fMRI experiment, they again investigated the perception of robotic and anthropomorphic movements performed by a robotic model and a digital human model. They presented to the subjects video clips with the four different types of movements (robot model—anthropomorphic vs. robotic movements; human model—anthropomorphic vs. robotic movements) in an MRI environment and investigated the underlying brain activity related to the observation of the different movements. Abel et al. (2022) were able to show that males and females use different processing pathways in the brain when processing the perception of robotic and anthropomorphic movements. Females showed activations of the left hemispheric primary and secondary visual areas while observing the robot model. Regarding the difference between anthropomorphic and robotic movements, female participants demonstrated significant activations in the right hemisphere in the primary sensory cortex, the superior parietal lobule, and the visual motor cortex. Male participants, however, showed activations in the movement coding areas in the posterior parieto-temporal cortex while observing the robot model and the robotic movements. All the activation sites (for female and for male) were localized in the dorsal stream of processing of visuomotor information (Goodale and Milner, 1992). In the classical view of Goodale & Milner the dorsal stream was a unified structure. Newer research has demonstrated that the dorsal stream is rather subdivided into the dorso-dorsal and ventro-dorsal substreams (Binkofski and Buxbaum, 2013). Along these lines, the activation sites in females were localized in the dorso-dorsal substream. The activation site in male, however, was localized in the ventro-dorsal substream. The two substreams of the dorsal stream of processing of the visuomotor information have distinct characteristics: the dorso-dorsal stream is processing online information and has a small working memory capacity, whereas the ventro-dorsal stream has much more working memory capacity and is using previous knowledge about actions in the action processing. The results of Abel et al. (2020) allow the following conclusions to be drawn (Abel et al., 2022): Females analyse different movements “online” via the dorso-dorsal stream, whereas males process these movements via the ventro-dorsal stream and use more working memory capacity and rely on their prior knowledge when analyzing movements. They seem to have a higher sensitivity to differences between robotic and anthropomorphic movements and employ their expert MNS for perception of robotic movements using their previous experiences in such situations.

These results are quite promising and shed first light on gender differences in perception of robotic movements. Nevertheless, further research is needed to get deeper insights into this issue.

## Conclusion

It can be stated that a working environment in which humans and robots interact with each other should be well thought out. More and more robots are being used to interact with humans, not only in industry but also in the healthcare sector. Hence, it is important to investigate how the respective acceptance of the robot is and which factors influence this. This can depend to large extent on the area of employment. There are various parameters that have an influence on the acceptance of human robot interaction. Besides the shape of a robot, the type of movement seems to be an important component. However, biological sex also seems to play a role in human robot interaction. It should be interesting to conduct further studies here to investigate the different influences, such as gender, previous experience, movement type, shape, and others, on human robot interaction in more detail. In particular, field studies that investigate human robot interaction in a real-world condition are of great relevance here. A laboratory condition, as in the studies described in this article, provide a foundation on which to build. In this context, it is also interesting to investigate the underlying neuroanatomical correlates. In addition to the MNS, the action observation system is also a relevant system which should be considered in more detail in the field of human robot interaction. In particular, fiber connections between neuroanatomical structures are also of interest here in order to better understand the systems and thus improve conditions in co-working scenarios between humans and robots. This can also be explored in field studies, e.g., with the utilization of portable functional near-infrared spectroscopy (fNIRS) technology.

## Author contributions

MA: Writing – original draft. GB: Writing – review & editing. FB: Conceptualization, Supervision, Writing – review & editing.

## References

[ref1] Abel M. Kuz S. Patel H. J. Petruck H. Klann J. Schlick C. M.. (2022). Anthropomorphic or non-anthropomorphic? Effects of biological sex in observation of actions in a digital human model and a gantry robot model. Front. Neurorobot. 16:937452. doi: 10.3389/fnbot.2022.937452, PMID: 36061147 PMC9428556

[ref2] Abel M. Kuz S. Patel H. J. Petruck H. Schlick C. M. Pellicano A.. (2020). Gender effects in observation of robotic and humanoid actions. Front. Psychol. 11:797. doi: 10.3389/fpsyg.2020.00797, PMID: 32425860 PMC7205409

[ref3] Bartneck C. Kulić D. Croft E. Zoghbi S. (2009). Measurement instruments for the anthropomorphism, animacy, likeability, perceived intelligence, and perceived safety of robots. Int. J. Soc. Robot. 1, 71–81. doi: 10.1007/s12369-008-0001-3

[ref4] Belanche D. Casaló L. V. Flavián C. Schepers J. (2020). Service robot implementation: a theoretical framework and research agenda. Serv. Ind. J. 40, 203–225. doi: 10.1080/02642069.2019.1672666

[ref5] Benitez J. Wyman A. B. Carpinella C. M. Stroessner S. J. (2017). The authority of appearance: how robot features influence trait inferences and evaluative responses. IEEE International Symposium on Robot and Human Inter Communication (RO–MAN)

[ref6] Binkofski F. Buxbaum L. J. (2013). Two action systems in the human brain. Brain Lang. 127, 222–229. doi: 10.1016/j.bandl.2012.07.007, PMID: 22889467 PMC4311762

[ref7] Buccino G. Colagè I. Gobbi N. Bonaccorso G. (2016). Grounding meaning in experience: a broad perspective on embodied language. Neurosci. Biobehav. Rev. 69, 69–78. doi: 10.1016/j.neubiorev.2016.07.033, PMID: 27477443

[ref8] Buccino G. Lui F. Canessa N. Patteri I. Lagravinese G. Benuzzi F.. (2004a). Neural circuits involved in the recognition of actions performed by non-conspecifics: an fMRI study. J. Cogn. Neurosci. 16, 114–126. doi: 10.1162/089892904322755601 15006041

[ref9] Buccino G. Vogt S. Ritzl A. Fink G. R. Zilles K. Freund H. J.. (2004b). Neural circuits underlying imitation learning of hand actions: an event-related fMRI study. Neuron 42, 323–334. doi: 10.1016/S0896-6273(04)00181-3, PMID: 15091346

[ref10] Calvo-Merino B. Glaser D. E. Grezes J. Passingham R. E. Haggard P. (2005). Action observation and acquired motor skills: an fMRI study with expert dancers. Cereb. Cortex 15, 1243–1249. doi: 10.1093/cercor/bhi007, PMID: 15616133

[ref11] Cameron D. Millings A. Fernando S. Collins E. C. Moore R. Sharkey A.. (2018). The effects of robot facial emotional expressions and gender on child–robot interaction in a field study. Connect. Sci. 30, 343–361. doi: 10.1080/09540091.2018.1454889

[ref12] Carpinella C. M. Wyman A. B. Perez M. A. Stroessner S. J. (2017). The robotic social attributes scale (RoSAS): development and validation. In: Proceedings of the 2017 ACM/IEEE Intern Conference on Human–Robot Interaction, 254–262.

[ref13] Chaminade T. Zecca M. Blakemore S. J. Takanishi A. Frith C. D. Micera S.. (2010). Brain response to a humanoid robot in areas implicated in the perception of human emotional gestures. PLoS One 5:e11577. doi: 10.1371/journal.pone.0011577, PMID: 20657777 PMC2908128

[ref14] Cross E. S. Liepelt R. Hamilton A. F. Parkinson J. Ramsey R. Stadler W.. (2012). Robotic movement preferentially engages the action observation network. Hum. Brain Mapp. 33, 2238–2254. doi: 10.1002/hbm.21361, PMID: 21898675 PMC6870135

[ref15] Di Pellegrino G. Fadiga L. Fogassi L. Gallese V. Rizzolatti G. (1992). Understanding motor events: a neurophysiological study. Exp. Brain Res. 91, 176–180. doi: 10.1007/bf00230027 1301372

[ref16] Dubois-Sage M. Jacquet B. Jamet F. Baratgin J. (2023). We do not anthropomorphize a robot based only on its cover: context matters too! Appl. Sci. 13:8743. doi: 10.3390/app13158743

[ref17] Duffy B. R. (2003). Anthropomorphism and the social robot. Special issue on socially interactive robots. Anthropomor. Autonom. Syst. 42, 177–190. doi: 10.1016/S0921-8890(02)00374-3

[ref18] Fogassi L. Ferrari P. F. Gesierich B. Rozzi S. Chersi F. Rizzolatti G. (2005). Parietal lobe: from action organization to intention understanding. Science 308, 662–667. doi: 10.1126/science.1106138, PMID: 15860620

[ref19] Gallese V. Fadiga L. Fogassi L. Rizzolatti G. (1996). Action recognition in the premotor cortex. Brain 119, 593–609. doi: 10.1093/brain/119.2.593 8800951

[ref20] Gallese V. Goldman A. (1998). Mirror neurons and the simulation theory of mindreading. Trends Cogn. Sci. 2, 493–501. doi: 10.1016/S1364-6613(98)01262-5, PMID: 21227300

[ref21] Gallese V. Keysers C. Rizzolatti G. (2004). A unifying view of the basis of social cognition. Trends Cogn. Sci. 8, 396–403. doi: 10.1016/j.tics.2004.07.002, PMID: 15350240

[ref22] Gazzola V. Rizzolatti G. Wicker B. Keysers C. (2007). The anthropomorphic brain: themirror neuron systemresponds to human and robotic actions. NeuroImage 35, 1674–1684. doi: 10.1016/j.neuroimage.2007.02.003, PMID: 17395490

[ref23] Goodale M. A. Milner A. D. (1992). Separate visual pathways for perception andaction. Trends Neurosci. 15, 20–25. doi: 10.1016/0166-2236(92)90344-8 1374953

[ref24] Hamilton A. F. Grafton S. T. (2008). Action outcomes are represented in human inferior frontoparietal cortex. Cereb. Cortex 18, 1160–1168. doi: 10.1093/cercor/bhm150, PMID: 17728264

[ref25] Hardwick R. M. Caspers S. Eickhoff S. B. Swinnen S. P. (2018). Neural correlates of action: comparing meta-analyses of imagery, observation, and execution. Neurosci. Biobehav. Rev. 94, 31–44. doi: 10.1016/j.neubiorev.2018.08.003, PMID: 30098990

[ref26] Haslinger B. Erhard P. Altenmuller E. Schroeder U. Boecker H. Ceballos-Baumann A. O. (2005). Transmodal sensorimotor networks during action observation inprofessional pianists. J. Cogn. Neurosci. 17, 282–293. doi: 10.1162/0898929053124893, PMID: 15811240

[ref27] Hoenen M. Lübke K. T. Pause B. M. (2016). Non-anthropomorphic robots as social entities on a neurophysiological level. Comput. Hum. Behav. 57, 182–186. doi: 10.1016/j.chb.2015.12.034

[ref28] Huber M. Rickert M. Knoll A. Brandt T. Glausauer S. (2008). “Human-robot interaction in handing-over tasks” in RO-MAN 2008 - the 17th IEEE international symposium on robot and human interactive communication (München: IEEE), 107–112.

[ref29] Hugues O. Weistroffer V. Paljic A. Fuchs P. Karim A. A. Gaudin T.. (2016). Determining the important subjective criteria in the perception of human-like robot movements using virtual reality. Int. J. Humanoid Anthropomor. 13:1550033. doi: 10.1142/S0219843615500334

[ref30] Karwowski W. (1991). “Human-robot interaction: an overview of perceptual aspects of working with industrial robots” in Towards human work: Solutions to problems in occupational health and safety. eds. Kumashiro M. Megaw E. D. (London: Taylor & Francis Group), 68–74.

[ref31] Kaysers C. Gazzola V. (2010). Social neuroscience: Mirror neurons recorded in humans. Curr. Biol. 20, R353–R354. doi: 10.1016/j.cub.2010.03.013 21749952

[ref32] Krach S. Hegel F. Wrede B. Sagerer G. Binkofski F. Kircher T. (2008). Interaction and perspective taking with robots investigated via fMRI. PLoS One 3:e2597. doi: 10.1371/journal.pone.0002597, PMID: 18612463 PMC2440351

[ref33] Kuz S. Mayer M. P. H. Müller S. Schlick C. M. (2013). “Using anthropomorphism to improve the human-machine interaction in industrial environments (part I)” in Digital Human Modeling and Applications in Health, Safety, Ergonomics, and Risk Management. Human Body Modeling and Ergonomics. ed. Duffy V. G. (Las Vegas, NV: Springer), 76–85.

[ref34] Lee E. J. (2008). Flattery may get computers somewhere, sometimes: the moderating role of output modality, computer gender, and user gender. Int. J. Hum. Comput. Stud. 66, 789–800. doi: 10.1016/j.ijhcs.2008.07.009

[ref35] Liepelt R. Prinz W. Brass M. (2010). When do we simulate non-human agents? Dissociating communicative and non-communicative actions. Cognition 115, 426–434. doi: 10.1016/j.cognition.2010.03.003, PMID: 20356576

[ref36] Lu L. Cai R. Gursoy D. (2019). Developing and validating a service robot integration willingness scale. Int. J. Hosp. Manag. 80, 36–51. doi: 10.1016/j.ijhm.2019.01.005

[ref37] Maier H. (2022). Grundlagen der Robotik (3. neu bearbeitete und erweiterte Auflage). Berlin, Germany: VDE Verlag GmbH.

[ref38] Michalos G. Karagiannis P. Dimitropoulos N. Andronas D. Makris S. (2022). “Human robot collaboration in industrial environments” in The 21st century industrial robot: *When tools become collaborators* (Cham: Springer), 17–39. doi: 10.1007/978-3-030-78513-0_2

[ref39] Mori M. (1970). The uncanny valley. Energy 7, 33–35.

[ref40] Mukamel R. Ekstrom A. D. Kaplan J. Iacoboni M. Fried I. (2010). Single-neuron responses in humans during execution and observation of actions. Curr. Biol. 20, 750–756. doi: 10.1016/j.cub.2010.02.045, PMID: 20381353 PMC2904852

[ref41] Ramsey R. Hamilton A. F. (2010). Triangles have goals too: understanding action representation in left aIPS. Neuropsychologia 48, 2773–2776. doi: 10.1016/j.neuropsychologia.2010.04.028, PMID: 20434468

[ref42] Rizzolatti G. Craighero L. (2004). The mirror neuron system. Annu. Rev. Neurosci. 27, 169–192. doi: 10.1146/annurev.neuro.27.070203.144230 15217330

[ref43] Savela N. Kaakinen M. Ellonen N. Oksanen A. (2021). Sharing a work team with robots: the negative effect of robot co-workers on in-group identification with the work team. Comput. Hum. Behav. 115:106585. doi: 10.1016/j.chb.2020.106585

[ref44] Seo S. (2022). When female (male) robot is talking to me: effect of service robots’ gender and anthropomorphism on customer satisfaction. Int. J. Hosp. Manag. 102:103166. doi: 10.1016/j.ijhm.2022.103166

[ref45] Stroessner S. J. Benitez J. (2019). The social perception of humanoid and non-humanoid robots: effects of gendered and machinelike features. Int. J. Soc. Robot. 11, 305–315. doi: 10.1007/s12369-018-0502-7

[ref46] Urgen B. A. (2015). Spatio-temporal neuroimaging of visual processing of human and robot actions in humans. (Doctoral dissertation). UC San Diego.

[ref47] Vogt S. Buccino G. Wohlschläger A. M. Canessa N. Shah N. J. Zilles K.. (2007). Prefrontal involvement in imitation learning of hand actions: effects of practice and expertise. NeuroImage 37, 1371–1383. doi: 10.1016/j.neuroimage.2007.07.005, PMID: 17698372

